# Rotation and torsion of the left ventricle with cardiovascular magnetic resonance tagging: comparison of two analysis methods

**DOI:** 10.1186/s12880-020-00473-4

**Published:** 2020-07-01

**Authors:** Lauri Lehmonen, Mikko Jalanko, Mika Tarkiainen, Touko Kaasalainen, Johanna Kuusisto, Kirsi Lauerma, Sauli Savolainen

**Affiliations:** 1 HUS Medical Imaging Center, Radiology, University of Helsinki and Helsinki University Hospital, PO Box 340, FI-00029 Helsinki, HUS Finland; 2 Department of Physics, University of Helsinki, Helsinki, Finland; 3 Department of Cardiology, Heart and Lung Center, Helsinki University Hospital and University of Helsinki, Helsinki, Finland; 4 Department of Clinical Radiology, Kuopio University Hospital, Kuopio, Finland; 5 Institute of Clinical Medicine, Internal Medicine and Kuopio University Hospital, Kuopio, Finland

**Keywords:** Cardiovascular magnetic resonance, Tagging, Rotation, Torsion

## Abstract

**Background:**

Left ventricle rotation and torsion are fundamental components of myocardial function, and several software packages have been developed for analysis of these components. The purpose of this study was to compare the suitability of two software packages with different technical principles for analysis of rotation and torsion of the left ventricle during systole.

**Methods:**

A group of hypertrophic cardiomyopathy (HCM) patients (*N* = 14, age 43 ± 11 years), mutation carriers without hypertrophy (*N* = 10, age 34 ± 13 years), and healthy relatives (*N* = 12, age 43 ± 17 years) underwent a cardiovascular magnetic resonance examination, including spatial modulation of magnetization tagging sequences in basal and apical planes of the left ventricle. The tagging images were analyzed offline using a harmonic phase image analysis method with Gabor filtering and a non-rigid registration-based free-form deformation technique. Left-ventricle rotation and torsion scores were obtained from end-diastole to end-systole with both software.

**Results:**

Analysis was successful in all cases with both software applications. End-systolic torsion values between the study groups were not statistically different with either software. End-systolic apical rotation, end-systolic basal rotation, and end-systolic torsion were consistently higher when analyzed with non-rigid registration than with harmonic phase-based analysis (*p* <  0.0001). End-systolic rotation and torsion values had significant correlations between the two software (*p* <  0.0001), most significant in the apical plane.

**Conclusions:**

When comparing absolute values of rotation and torsion between different individuals, software-specific reference values are required. Harmonic phase flow with Gabor filtering and non-rigid registration-based methods can both be used reliably in the analysis of systolic rotation and torsion patterns of the left ventricle.

## Background

Motion of the heart is a result of the order of the arrangement of myofibers in the heart wall. The myofibers are organized into a helical wrap around the left ventricle, with a gradual change in the fiber orientation from a right-handed helix in the subendocardial layers to a left-handed helix in the epicardial layers [1]. As the myofibers contract, the ventricular wall contracts, thickens, and rotates in different planes of the heart. During systole the myofibers create a rotating motion, pushing blood forward and reducing the volume of the left ventricle. When looking at the heart from the apex, this wringing motion is created by a clockwise basal rotation and a counterclockwise apical rotation [2]. Different diseases damage the heart and weaken its motion.

Quantitative analysis is increasingly popular to allow finer distinctions between healthy and diseased tissue and to compare results of different studies more reliably. Cardiovascular magnetic resonance (CMR) tagging is a method for creating temporary visually detectable saturation patterns (stripes or grids) in CMR images. The tagging pattern is created using the same magnetic properties of tissue that are used for image acquisition. During the pulsating motion of the heart the tagging pattern deforms as the heart moves, and by analyzing this deformation, it is possible to determine myocardial motion. Several other imaging methods, such as computed tomography, single photon emission tomography, and positron emission tomography, have also been used for motion analysis of the heart [3, 4]. The most recent developments have centered around use of ultrasonic methods [5]. Despite advancements, CMR remains the most consistent method and is considered the gold standard in myocardial motion assessment [6, 7].

The technique for CMR tagging has existed for over 20 years; however, in clinical work the method is still not widely used due to the lack of easily accessible tools for analysis. Many available analysis tools started as research projects and have later been commercialized. The goal of this work was to investigate the suitability of two available software packages and to compare the results yielded by them in three different study groups: hypertrophic cardiomyopathy (HCM) patients, mutation carriers without hypertrophy, and healthy controls. In HCM, the ventricular wall is asymmetrically thickened, leading to local dysfunction and a change in contractile mechanics. HCM has been employed in this study as a suitable disease model to assess the usefulness of analysis tools for myocardial mechanics.

## Methods

### Study population

The study population was selected from a previous study of myosin-binding protein C gene (MYBPC), which consisted of 32 patients carrying the Finnish founder mutation in the MYBPC gene (MYBPC3-Q1061X) with left-ventricle hypertrophy consistent with HCM phenotype (left-ventricular maximal wall thickness > 13 mm in CMR), 15 subjects with the mutation and no HCM phenotype (left-ventricular maximal wall thickness < 13 mm in CMR), and their 20 healthy relatives without the mutation [8]. Genetic diagnosis was performed on all subjects [9]. Of these individuals, 36 (14 HCM patients, 10 mutation carriers, and 12 healthy relatives) had undergone CMR with tagging images acquired and were selected for this study. The study protocol was approved by the Ethics Review Board of Helsinki and Uusimaa, and a written informed consent was received from all participants. All of the individuals included in the study underwent a CMR examination between 2009 and 2011 using a Magnetom Avanto 1.5 T system (Siemens Healthcare, Erlangen, Germany). Six-channel body and six-channel spine coils were used for the acquisition. Prospective ECG-gating and breath-hold were used in all cases to minimize acquisition problems related to arrhythmia and to minimize motion artifacts caused by breathing. The CMR imaging protocol included typical volumetric assessments as well as tagging sequences in basal and apical planes of the left ventricle.

### CMR tagging

In this study, the tagging images were acquired at apical and basal levels of the left ventricle. Different technical solutions exist for the creation of the tagging pattern [10]. The pulse sequence used in all cases in this study was a typical spatial modulation of magnetization tagging sequence [11] provided by the vendor of the magnetic resonance imaging system. The sequence had a grid tagging pattern, 8 mm slice thickness, 8 mm distance between the tagging lines, repetition time of 41 ms, echo time of 4.0 ms, 14° flip angle, matrix size of 208 × 256, and voxel size of 1.25 × 1.25 × 8 mm^3^. The sequence had 20–25 temporal phases.

### Image analysis

The analysis of the apical and basal tagging images was performed using two different software, Harmonic Phase Flow (HPF) plugin (Computer Vision Center, Barcelona, Spain) [12–14] for Osirix Dicom viewer v7.0.2 (Pixmeo, Geneva, Switzerland) and Segment strain tagging module v2.2 R6190 (Medviso AB, Lund, Sweden) [15, 16]. The two software use different technical approaches in analysis of tagging images. Both software required manual epicardial and endocardial segmentation in a single time frame in the plane of analysis.

One way of analyzing the deformation of the tagging pattern is harmonic phase (HARP) analysis [17]. In HARP analysis, the intensity of a tagged CMR image is expressed in complex form in the Fourier space. HARP images are only a material quantity and they remain constant over time. Therefore, they can be used to follow the location of the tagging pattern through the cardiac cycle and to construct a deformation map. The motion following of HARP images is an image-processing problem affected by image quality [18]. HPF solves the HARP tracking problem using a Gabor filter bank in an alternative mathematical framework. Optimal results using Gabor filters can be achieved when using different filter parameters in each plane of the left ventricle since the motion of the heart is different in each plane. The motion of a single myocardial point can be quantified by analyzing its motion through the imaging sequence. Rotation is defined as the relative change of an angle θ between the time points zero and t. The location of a single point of the myocardium at time point t + 1 is obtained by operating it with a motion vector calculated at time point t. The motion of the entire left ventricle is calculated by forming a continuous motion vector (or deformation map) over the imaging sequence. Rotation of the entire left ventricle in a single plane is calculated as a normalized scalar product with respect to the center of mass of the ventricle [19]:

1 $$ \mathrm{R}\left(\uptheta \right)=\operatorname{arccos}\frac{\left\langle {\mathrm{P}}_0-{\mathrm{C}}_0,{\mathrm{P}}_{\mathrm{t}}-{\mathrm{C}}_{\mathrm{t}}\right\rangle }{\left|\left|{\mathrm{P}}_0-{\mathrm{C}}_0\right|\right|\left|\left|{\mathrm{P}}_{\mathrm{t}}-{\mathrm{C}}_{\mathrm{t}}\right|\right|} $$

where arccos is the inverse of cosine, and the numerator denotes a vector product between vectors P_0_ − C_0_ and P_t_ − C_t_. P_0_ and P_t_ are the locations of a single myocardial point at the time points zero and t. C_0_ and C_t_ are the locations of the center of mass of the left ventricle at time points zero and t. The denominator is a normalized scalar product of the same vectors. Torsion is then defined as the rotation difference between the apical and basal levels of the left ventricle.

Segment strain tagging module approaches the tagging pattern analysis in image space, working with non-rigid elastic image registration. The tagging module maps the displacement of single myocardial points between consecutive time frames and presents the displacements of all myocardial points as two-dimensional third-order B-spline tensor products [20]. An inter-frame transformation field is then constructed using a limited memory Broyden-Fletcher-Goldfarb-Shannon optimizer. This transformation field can be used to calculate strains, velocities, and displacements between end-diastole and end-systole of the left ventricle. To solve the displacement or rotation of the left-ventricle wall, cumulating the transformation field through all time frames is required [16]. Torsion in Segment software is defined as the difference between apical and basal rotation, normalized with the distance between the two slices, and the mean radius of the heart wall in these slices. Torsion was calculated in the same way as in HPF for better comparison between the two software.

Results of rotation and torsion data were collected, with respect to time, from end-diastole to end-systole with both software, and exported to MATLAB R2019A (The MathWorks, Inc., Natick, MA, USA) for visualization. Additionally, end-systolic values were collected. HPF reports the relative time as a proportion from 0% (end-diastole) to 100% (end-systole), while Segment uses an absolute time scale. To visually compare rotation curves derived with each software, Segment curves were manually converted in MATLAB to the same 0–100% time scale as in HPF.

### Statistical analysis and intraobserver variability

Results are expressed as mean ± standard deviation. Statistical analysis was performed using IBM SPSS Statistics 25 for Windows (IBM Corp., Armonk, NY, USA). Independent samples Kruskal-Wallis test was used to test for significant differences in volumetric parameters and tagging results, and Spearman’s rho was used to test for correlations between rotation and torsion values obtained with the two software. *P*-values less than 0.05 were considered statistically significant. Only end-systolic values were included in the statistical analysis. Bland-Altman method was used to assess intraobserver variability in the tagging results with both software, and all study subjects were selected for the analysis. Mean differences (bias) and 95% limits of agreement (±1.96 standard deviations) were computed.

## Results

### Volumetric data

Baseline clinical information and volumetric data are reported in Table 1. There were more males in the HCM group and no differences in left-ventricular volumes or ejection fractions between the groups. As expected, the left-ventricular mass was significantly higher in the HCM group.

**Table 1:** Basic clinical features and volumetric data of each group. Data are presented as mean ± standard deviation

Parameter	HCM *N* = 14	Mutation *N* = 10	Healthy *N* = 12	*p*-value
Number of females (proportion)	4 (25%)	10 (100%)	8 (75%)	–
Age (years)	43 ± 11	34 ± 13	43 ± 17	0.280
Height (cm)	177 ± 9	166 ± 4	169 ± 9	0.015*
Weight (kg)	80 ± 15	60 ± 3	79 ± 11	0.005*
BSA (m^2^)	2.0 ± 0.7	1.7 ± 0.0	1.8 ± 0.3	0.003*
LVEF (%)	63 ± 8	62 ± 6	60 ± 9	0.225
LVEDVI (ml/ m^2^)	81 ± 11	77 ± 13	79 ± 12	0.819
LVESVI (ml/ m^2^)	30 ± 10	29 ± 7	32 ± 11	0.625
LVMI (g/ m^2^)	60 ± 18	42 ± 7	42 ± 8	0.005*
Max LV wall thickness (mm)	22 ± 7	9 ± 1	10 ± 3	< 0.0001*
Distance between tagging planes (cm)	4.0 ± 0.5	3.9 ± 0.3	3.7 ± 0.2	0.098

*BMI* Body mass index, *BSA* Body surface area, *LV* Left-ventricle, *EF* Ejection fraction, *EDVI* End-diastolic volume indexed, *ESVI* End-systolic volume indexed, *MI* Mass indexed, * statistically significant (*p* < 0.050)

### Rotation and torsion

The mean analysis time per study was 4 ± 2 min in HPF and 5 ± 2 min in Segment. All cases were applicable for analysis with both software. End-systolic peak apical rotation, peak basal rotation, and peak torsion values were collected (Table 2). HPF showed constantly smaller values than Segment. Looking at the end-systolic rotation and torsion values obtained with different software, similar observations were detected in the study groups. End-systolic apical rotation was smallest in the HCM group (HPF: 1.8° ± 1.8°; Segment: 3.4° ± 4.5°), and end-systolic torsion was largest in the mutation group (HPF: 4.1° ± 1.6°; Segment: 9.5° ± 2.2°). The end-systolic rotation or torsion values were not statistically different between the study groups with either software.

**Table 2:** Results for end-systolic rotation and torsion in all study groups

Parameter	HCM *N* = 14	Mutation *N* = 10	Healthy *N* = 12	*p*-value
HPF apical rotation (°)	1.8 ± 1.8	3.0 ± 1.7	1.9 ± 1.9	0.266
HPF basal rotation (°)	−1.5 ± 1.2	−1.1 ± 0.9	−1.9 ± 1.3	0.351
HPF torsion (°)	3.3 ± 1.7	4.1 ± 1.6	3.8 ± 1.3	0.358
Segment apical rotation (°)	3.4 ± 4.5	6.0 ± 2.3	4.0 ± 4.3	0.457
Segment basal rotation (°)	−3.8 ± 2.5	−3.6 ± 2.3	−4.0 ± 2.5	0.667
Segment torsion (°)	7.2 ± 3.7	9.5 ± 2.2	8.0 ± 2.6	0.154
Segment torsion, normalized (°/mm)	0.17 ± 0.07	0.22 ± 0.07	0.22 ± 0.05	0.195

*HCM* Hypertrophic cardiomyopathy, *HPF* Harmonic Phase Flow

**Fig. 1:**
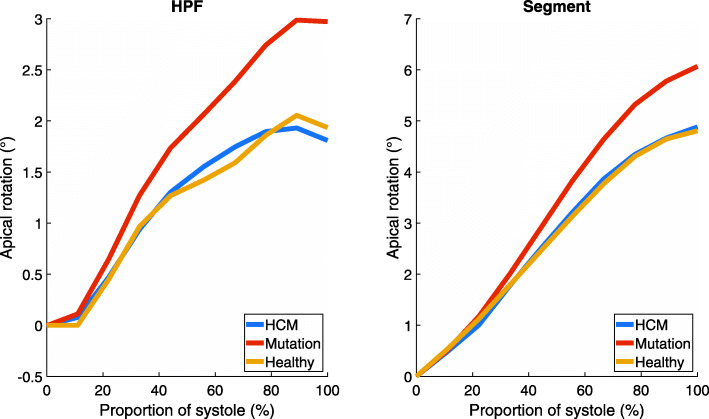
Mean apical rotation in each study group

**Fig. 2:**
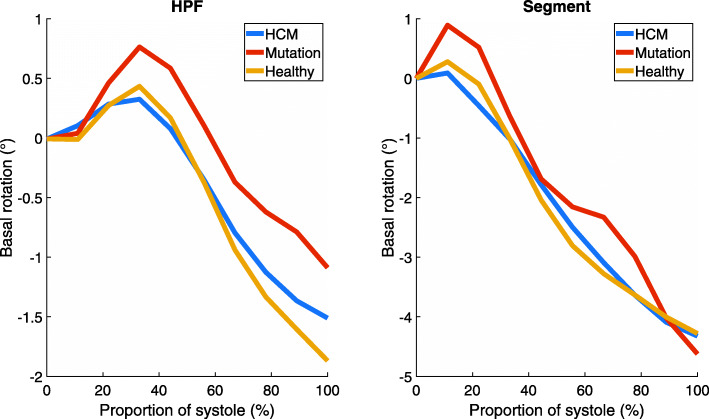
Mean basal rotation in each study group

**Fig. 3:**
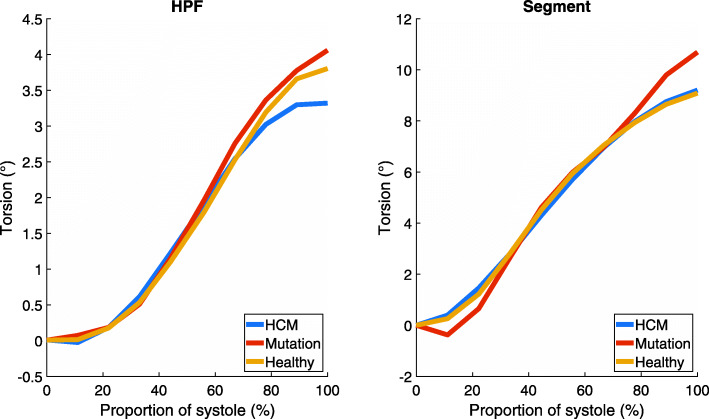
Mean torsion of the left ventricle in each study group

The rotation curves of HCM patients and healthy controls followed similar paths in both apical and basal planes of the left ventricle (Figs. 1 and 2). The largest difference between curves in HPF and Segment was seen in basal rotation in the mutation group (Fig. 2). The other curves agreed well between HPF and Segment. In the apical plane, the end-systolic rotation was higher in mutation carriers than in HCM patients or healthy controls. The rotation curves of mutation carriers were separated from the rotation curves of the other groups in both apical and basal planes. Apical rotation was stronger throughout systole. In Fig. 2, the initial positive basal rotation of mutation carriers was higher than that of healthy controls or HCM patients. Torsion curves of different study groups (Fig. 3) look similar throughout systole.

**Fig. 4:**
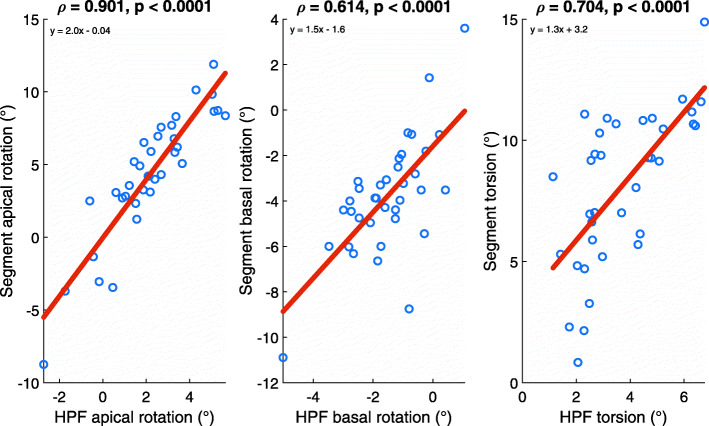
Correlations between end-systolic rotation and torsion values obtained with and Harmonic Phase Flow (HPF) and Segment

Absolute rotation and torsion values were significantly different in HPF compared with the respective values in Segment software (*p* < 0.0001). These values were considerably higher in Segment. Statistically significant correlations were detected in apical rotation, basal rotation, and torsion values between these two software (Fig. 4). The correlation in apical rotation was the strongest (*p* > 0.9).

### Intraobserver variability

**Fig. 5:**
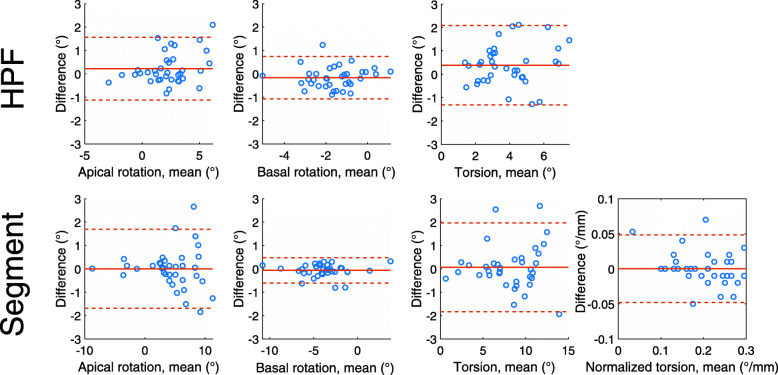
Intraobserver variability of rotation and torsion results with Harmonic Phase Flow (HPF) and Segment. Solid lines in each graph denote bias and dashed lines denote 95% limits of agreement

Bland-Altman plots for both software packages are presented in Fig. 5. Both software packages showed good intraobserver reliability. With HPF, 97% of data points were within the 95% confidence interval, compared to 92% with Segment. Basal rotation showed smaller variability compared to apical rotation with both software. Segment showed slightly smaller bias between two measurements compared to HPF.

## Discussion

The aim of our study was to evaluate the suitability of HPF and Segment software in analyzing rotation and torsion of the left ventricle in different study groups. This is the first study to compare differences between these two software packages. Analysis was successful in all cases and usage of both software was robust. Absolute end-systolic rotation and torsion values differed significantly between the two software, Segment yielding systematically larger values. Software-specific reference values are required when comparing absolute values of rotation and torsion between different software. Correlations between the rotation and torsion values obtained with HPF and Segment were statistically significant, the most so in the apical plane.

Based on the results of both software, absolute torsion of the left ventricle did not differ significantly between HCM patients, mutation carriers, and healthy controls. Rotation of the apical level of the left ventricle was increased in mutation carriers. By contrast, basal rotation was slightly lower in this group (Fig. 2). As torsion is calculated as the angle difference between apical and basal rotations, the differences in rotation are not seen in the torsion curves (Fig. 3). This could indicate that HCM mutation without actual hypertrophy might already affect the motion of the left ventricle. However, the sample size is too small to draw definitive conclusions.

The effects of HCM on the motion of the left ventricle have been investigated before, but, as He et al. [21] summarized, the effects of location and extent of hypertrophy on left ventricular function have not attracted much attention, and further studies should be conducted to deepen understanding of disease mechanics. Previous studies have shown that peak torsion in HCM patients can be slightly higher than in control groups [22]. However, in the case of apical hypertrophic cardiomyopathy, apical rotation can be markedly decreased, while basal rotation is preserved [23]. In three of the HCM patients in our study, end-systolic apical rotation was negative, leading to an impaired torsion value. As HCM is a heterogeneous disease with diverse effects on the motion of the heart, more work is needed to elucidate the mechanics of the HCM heart.

Previous studies by Rüssel et al. [24] indicate end-systolic torsion in healthy subjects between basal and apical levels of the left ventricle of 7.7° ± 1.4°. Our result with Segment was similar (8.0° ± 2.6°). However, the result of the HPF was significantly lower (3.8° ± 1.3°). The definition for torsion applied by Rüssel et al. uses the mean radius of apical and basal levels divided by the mean distance between the two planes to normalize the torsion between different-sized hearts. The definition used by HPF for torsion in our work is absolute and does not take into account the size of the heart.

Gabor filter-based motion tracking has been previously shown to result in accurate segmentation of the tagging lines [25]. Gabor filtering achieves optimal locating in both spatial and Fourier domains, making it more suitable for tag line motion analysis than pure HARP [26]. The performance of the HPF algorithm has been assessed by the developers using a computational model as well as clinical data. They concluded that with sufficient image quality, HPF tracks motion correctly within sub-pixel accuracy [12]. Similarly, Segment strain tagging module has been validated by the developers, and the underlying non-rigid elastic registration-based motion analysis algorithm has been shown to yield clinically reproducible results between different observers with varying level of training [16, 27]. Our intraobserver analysis of the present study is in line with previous results.

Apical tagging imaging plane should be chosen carefully, as apical rotation is highly dependent on the imaging plane. The generally lower torsion values of HPF in our study could be due to the apical plane being close to mid-level of the left ventricle. Increased end-systolic apical rotation and end-systolic torsion values were detected in subjects with longer distance between the tagging planes.

### Limitations

Our study is limited to the rotation and torsion analyses of systole only, and readers were not blinded to the diagnosis of different cases. Interobserver variability was assessed in the present study. The patient sample of this study, being a subsample of a previous study, is too small to allow definitive conclusions to be drawn regarding the acquired rotation and torsion values between HCM patients, mutation carriers, and their healthy relatives.

## Conclusions

When comparing absolute values of CMR-derived rotation and torsion between different individuals, software-specific reference values are required. Harmonic Phase Flow OsiriX plugin and Segment strain tagging module can both be used to evaluate rotation and torsion patterns during systole, but absolute values between these software are significantly different.

## Data Availability

The datasets generated and/or analysed during the current study are not publicly available due to the limitations set by the ethical approval for this study. The datasets generated during this study are available from the corresponding author on reasonable request.

## Abbreviations

CMR: Cardiovascular magnetic resonance
HARP: Harmonic phase
HCM: Hypertrophic cardiomyopathy
HPF: Harmonic Phase Flow
MYBPC: Myosin-binding protein C gene
